# Comprehensive Prosthodontic Rehabilitation of a Completely Edentulous Adolescent With Cleft Lip and Palate and Multiple Facial Defects: A Case Report

**DOI:** 10.7759/cureus.111219

**Published:** 2026-06-20

**Authors:** Sai Prathyusha Yarra, Narendra Reddi, Mahalakshmi Gujjalapudi, Srujana Zakkula, Pragnyatha Pavani Nerella

**Affiliations:** 1 Department of Prosthodontics, Government Dental College and Hospital, Vijayawada, IND

**Keywords:** cleft lip and palate, complete denture, craniofacial abnormalities, edentulism, maxillofacial prosthesis, microtia, silicones

## Abstract

Cleft lip and palate (CLP) is the most prevalent congenital craniofacial anomaly and is frequently associated with dental anomalies and compromised maxillary anatomy. Complete edentulism in adolescents with CLP is exceedingly uncommon, and its concurrent presentation with bilateral auricular agenesis and nasal hypoplasia represents a rare clinical scenario. A 17-year-old male patient with surgically corrected bilateral CLP, congenital bilateral auricular agenesis, and a congenital nasal defect presented with complete edentulism of both arches. Comprehensive prosthodontic rehabilitation was performed. Intraoral restoration was accomplished using conventional heat-polymerized acrylic resin complete dentures, fabricated following meticulous border molding, definitive elastomeric impressions, and semiadjustable articulator mounting. Extraoral defects were restored using room-temperature vulcanizing silicone prostheses: a spectacle-frame-retained bilateral auricular prosthesis fabricated via a three-piece mold design to accommodate complex anatomical undercuts, and an adhesive-retained nasal prosthesis fabricated via a two-piece mold with putty protection of the nasal apertures. Intrinsic colorimetric matching was achieved using a systematic pigment incorporation technique. At the one-, three-, six-, and 12-month follow-up visits, the patient and guardian reported improvements in mastication, speech, social confidence, and overall satisfaction with the rehabilitation. This case illustrates that a structured, multidisciplinary prosthodontic approach integrating conventional complete dentures with custom-fabricated silicone maxillofacial prostheses can reliably restore orofacial function and esthetics in young patients with complex congenital craniofacial deficits, including those in resource-limited clinical settings.

## Introduction

Cleft lip and palate (CLP) is the most common congenital craniofacial malformation, affecting approximately 1.38 per 1,000 births in low- and middle-income countries, with notable variations across geographic and ethnic populations [1]. This condition results from failed fusion of the facial processes during embryonic development and is frequently associated with a spectrum of structural consequences, including maxillary hypoplasia, palatal scarring, dental agenesis, alveolar deficiency, and disturbances in speech and hearing [2]. Lifelong multidisciplinary management encompassing surgical correction, orthodontic therapy, speech rehabilitation, and dental restoration is typically required to optimize functional and esthetic outcomes in affected individuals [3].

Among the dental sequelae of CLP, complete edentulism during adolescence is a particularly rare occurrence [4]. When it does arise, the combination of a deficient maxillary arch, residual scar tissue from prior surgical interventions, and anatomically compromised denture-bearing tissues renders conventional complete denture fabrication considerably more challenging than in standard edentulous presentations. The shallow palatal vault, altered tissue resilience, and reduced alveolar support collectively impair prosthesis retention, stability, and occlusal equilibration [5].

The concurrent presentation of complete edentulism with bilateral auricular agenesis and nasal hypoplasia in a patient compounds the clinical complexity substantially. Auricular prostheses, whether retained by adhesives, osseointegrated implants, or spectacle frames, have been documented individually in the literature for both acquired and congenital defects [6,7]. Similarly, nasal prostheses fabricated using silicone elastomers have been reported in isolation [8]. However, to the best of our knowledge, the integrated management of all three deficits simultaneously, such as complete intraoral edentulism, bilateral auricular absence, and nasal hypoplasia in a single young patient with CLP, has not been previously described.

This case report presents the comprehensive prosthodontic rehabilitation of a 17-year-old completely edentulous male patient with surgically corrected bilateral CLP, congenital bilateral auricular agenesis, and a congenital nasal defect. This report details the clinical rationale, step-by-step treatment sequence, materials employed, and short-term functional and esthetic outcomes, with the intent of providing a reproducible reference for clinicians encountering similarly complex congenital presentations, particularly in settings where advanced digital fabrication technologies may be unavailable.

## Case presentation

A 17-year-old male patient of Indian ethnicity was referred to the Department of Prosthodontics, Government Dental College and Hospital, Vijayawada, India, in March 2025 with primary complaints of difficulty in mastication, difficulty with speech articulation, significant esthetic concerns attributable to complete tooth loss, bilateral auricular agenesis, and a malformed nose. Additionally, the patient and his guardian reported marked social withdrawal and psychological distress precipitated by his facial appearance, with avoidance of peer interactions and educational settings. Written informed consent has been obtained from the parent for the use of his records in publication.

The patient's birth history was notable for the diagnosis of three concurrent congenital anomalies: bilateral complete CLP, which was surgically corrected at six months of age via primary cheiloplasty and palatoplasty; bilateral auricular agenesis (Grade IV microtia), characterized by complete absence of external ear structures with preservation of small soft tissue vestiges in the auricular regions; and a congenital nasal defect manifesting as severe underdevelopment of the nasal alae, a depressed nasal bridge, and absence of the columella. No additional systemic comorbidities were noted. Family history was noncontributory to congenital craniofacial anomalies. Given the coexistence of multiple congenital craniofacial anomalies, genetic counseling and chromosomal evaluation were recommended; however, these investigations could not be pursued because of financial and resource limitations. With regard to dental history, the patient had been completely edentulous across both arches for approximately four years prior to presentation, attributable to exfoliation of the primary dentition without subsequent eruption of permanent successors. The precise etiopathological basis of eruption failure could not be definitively established based on the available clinical history. The patient had no prior experience of using any form of dental prosthesis.

**Figure 1:**
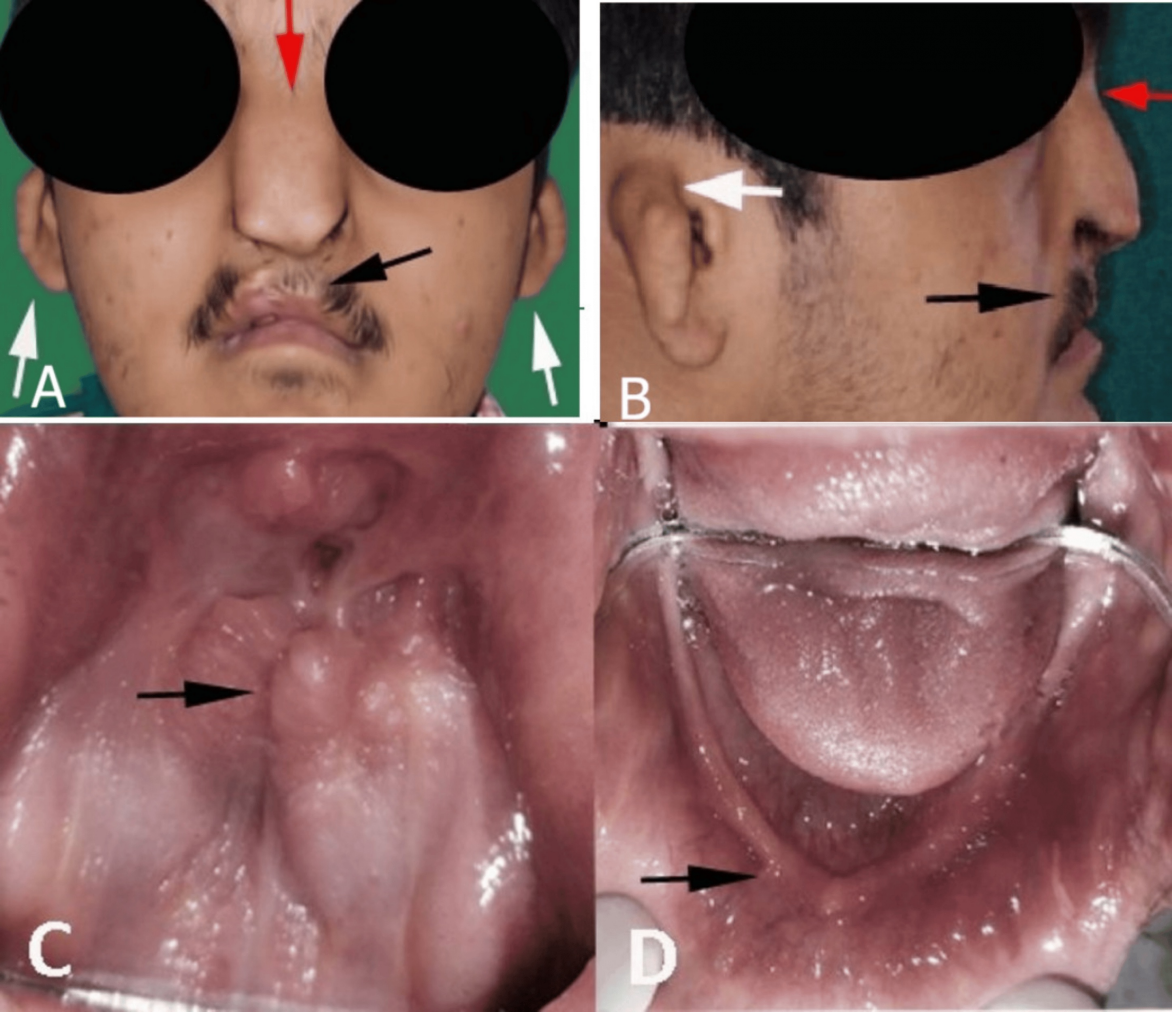
Clinical features. (A) Frontal view showing flat nasal bridge (red arrow), auricular defect (white arrows), and maxillary hypoplasia (black arrow). (B) Side view showing nasal defect (red arrow), auricular defect (white arrow), and maxillary hypoplasia (black arrow). (C) Edentulous maxillary arch with midline cleft (black arrow). (D) Edentulous mandibular arch (black arrow)

Extraoral examination demonstrated complete bilateral auricular agenesis, with only rudimentary soft tissue remnants identifiable in the preauricular regions. The nasal architecture was severely deficient, characterized by a markedly depressed and flattened nasal bridge, hypoplastic nasal alae, and complete absence of the columella. Midfacial retrusion, consistent with known CLP history and previous surgical correction, was evident on frontal and lateral evaluations. No oronasal fistulas were clinically detected. Facial asymmetry was noted on clinical examination (Figures 1A, 1B).

Intraoral evaluation revealed complete edentulism in the maxillary and mandibular arches. The maxillary denture-bearing area demonstrated a shallow palatal vault with midline scar tissue resulting from the prior palatoplasty. The scar contracture was palpable, although no oronasal fistula was evident (Figure 1C). The mandibular arch presented with no mucosal pathology (Figure 1D). The oral mucosa across both arches was healthy and free of inflammatory signs. Tongue morphology and mobility were within normal limits.

**Figure 2:**
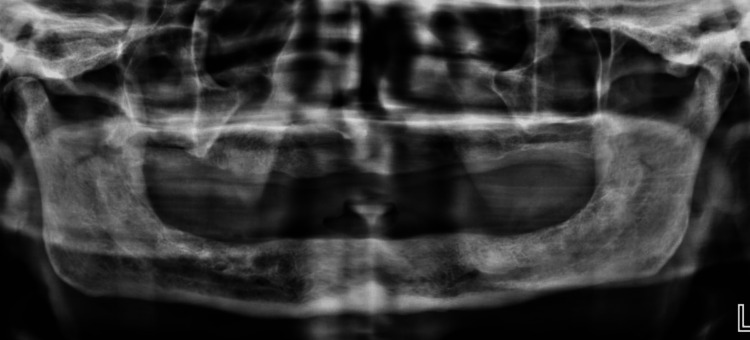
Orthopantogram showing edentulous status of maxillary and mandible jaw

Temporomandibular joint assessment revealed a symmetrical pain-free range of mandibular motion, without joint sounds. The masticatory musculature was nontender on bilateral palpation. Advanced diagnostic imaging such as cone-beam computed tomography was not performed owing to financial constraints and the patient's age; clinical evaluation was deemed sufficient to confirm the completely edentulous status. The edentulous status was further confirmed by panoramic radiograph (Figure 2). Panoramic radiographic examination revealed complete absence of developing permanent tooth buds in both the maxillary and mandibular arches, confirming the clinical finding of complete edentulism. The alveolar ridges demonstrated generalized reduction in height, which was more pronounced in the maxilla, although sufficient residual bone morphology was present to support conventional prosthodontic rehabilitation. No impacted teeth, retained root fragments, cystic lesions, or other residual osseous pathology were identified.

The patient had not previously received prosthodontic rehabilitation primarily because of the unusual and highly complex nature of his congenital craniofacial anomalies, combined with financial and resource limitations. Complete edentulism in adolescence is itself rare, particularly in association with CLP and multiple facial defects, making treatment planning more challenging than routine prosthodontic cases. Additionally, the patient had ongoing craniofacial growth, significant anatomical deficiencies, and no prior access to specialized multidisciplinary prosthodontic care. The absence of permanent tooth eruption resulted in complete edentulism, which significantly compromised oral function and esthetics and ultimately necessitated comprehensive prosthetic rehabilitation at the present stage. Rehabilitation was structured across 11 sequential phases spanning 11 weeks, as summarized in Table 1.

**Table 1:** Sequential treatment timeline for comprehensive prosthodontic rehabilitation RTV: room-temperature vulcanizing

Phase	Procedure	Time from initial visit
I	Preliminary examination, medical history, diagnostic impressions, photographic documentation	Day 1
II	Primary impressions (maxillary, mandibular, auricular, nasal); primary cast fabrication; custom tray construction	Weeks 1 and 2
III	Border molding; definitive impressions (light-body elastomeric); master cast fabrication	Week 3
IV	Record base fabrication; maxillomandibular relation registration; occlusal rim construction; articulator mounting	Week 4
V	Tooth arrangement; wax try-in; evaluation of esthetics, phonetics, and occlusion	Week 5
VI	Auricular and nasal wax pattern fabrication; combined extraoral wax try-in	Weeks 6 and 7
VII	Denture processing (heat-polymerized acrylic resin); extraoral mold flasking (three-piece for auricular, two-piece for nasal)	Week 8
VIII	RTV silicone packing with intrinsic pigmentation; polymerization (24 hours)	Week 9
IX	Finishing and polishing of dentures; retrieval and trimming of extraoral prostheses	Week 10
X	Final insertion of complete dentures and extraoral prostheses; patient and guardian education	Week 11
XI	Follow-up appointments at 1, 3, 6, and 12 months postinsertion	Ongoing

Primary impressions and cast fabrication

**Figure 3:**
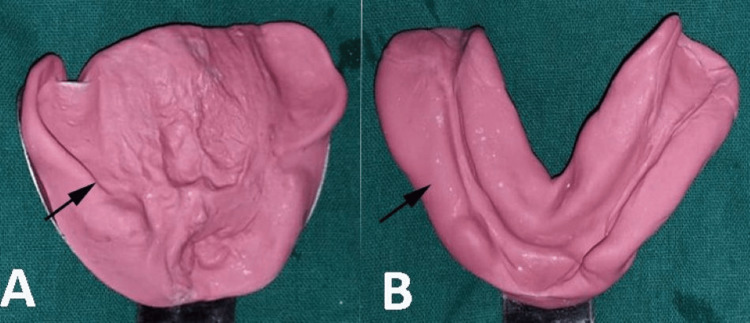
Primary impression of the (A) maxillary edentulous jaw (black arrow) and (B) mandibular edentulous jaw (black arrow)

Primary impressions of the completely edentulous maxillary and mandibular arches were obtained using a Type I impression compound (DPI Pinnacle Impression Compound; Dental Products of India Pvt. Ltd., Mumbai, India). The impressions were poured into plaster of Paris under standardized conditions to obtain primary diagnostic casts, which were subsequently retrieved, trimmed, and evaluated for anatomical accuracy (Figures 3A, 3B).

Custom tray construction and definitive impressions

**Figure 4:**
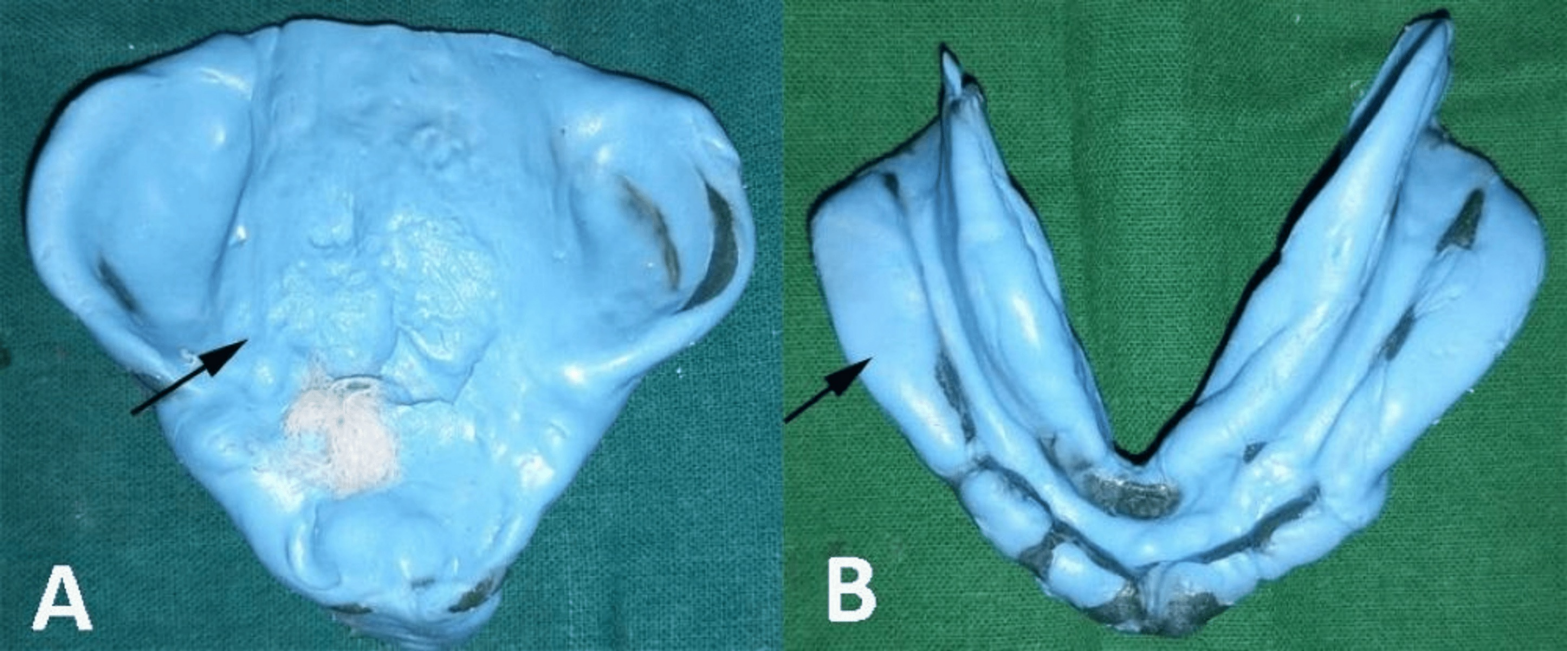
Definitive impressions recorded using light-body polyvinyl siloxane impression material. (A) Maxillary impression (black arrow). (B) Mandibular impression (black arrow)

Individual custom trays were fabricated on primary casts using autopolymerizing acrylic resin (DPI Self-Cure Acrylic Resin; Dental Products of India Pvt. Ltd., Mumbai, India). Peripheral border molding was performed using a green stick thermoplastic compound (DPI Green Stick Compound; Dental Products of India Pvt. Ltd., Mumbai, India) to achieve an optimal peripheral seal and flange extension. Definitive impressions were recorded using light-body polyvinyl siloxane impression material (Aquasil LV; Dentsply Sirona, Charlotte, NC, USA) for precise tissue reproduction (Figures 4A, 4B). The impressions were poured into Type III dental stone (Kalrock; Kalabhai Karson Pvt. Ltd., Mumbai, India) to obtain the master casts.

Maxillomandibular relations and tooth arrangement

Record bases were fabricated using autopolymerizing acrylic resin, and occlusal rims were constructed for registration of maxillomandibular relations. The vertical dimension of the occlusion was established using standard cephalometric and phonetic guidelines. The casts were mounted on a semiadjustable articulator, and the teeth were arranged using prefabricated acrylic denture teeth (Acry Rock; Ruthinium Group, Badia Polesine, Italy). Esthetic, phonetic, and occlusal parameters were evaluated during the wax try-in stage, and necessary modifications were made prior to processing.

Processing and delivery

Following patient approval of the wax trial dentures, the prostheses were processed using heat-polymerized acrylic resin through conventional flasking, dewaxing, and compression molding procedures. After bench cooling, the dentures were retrieved, finished on appropriate laboratory burs and polishing wheels, and polished to a high luster. Final denture insertion was preceded by careful occlusal verification and pressure-point identification.

Defect impressions and working casts

**Figure 5:**
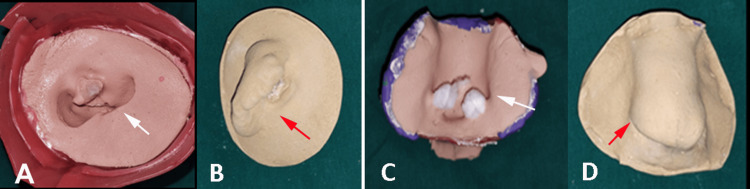
Impression of defects and cast fabrication. (A) Auricular defect impression (white arrow). (B) Auricular defect cast (red arrow). (C) Nasal defect impression (white arrow). (D) Nasal defect cast (red arrow)

Extraoral impressions of the bilateral auricular (Figure 5A) and nasal defects (Figure 5C) were obtained using an irreversible hydrocolloid impression material (Algitex; Dental Products of India Pvt. Ltd., Mumbai, India). The impressions were immediately poured into a Type IV die stone (Kalabhai Ultra Rock Die; Kalabhai Karson Pvt. Ltd., Mumbai, India) to obtain dimensionally accurate working casts for subsequent prosthetic sculpting (Figures 5B, 5D).

Auricular wax pattern fabrication

**Figure 6:**
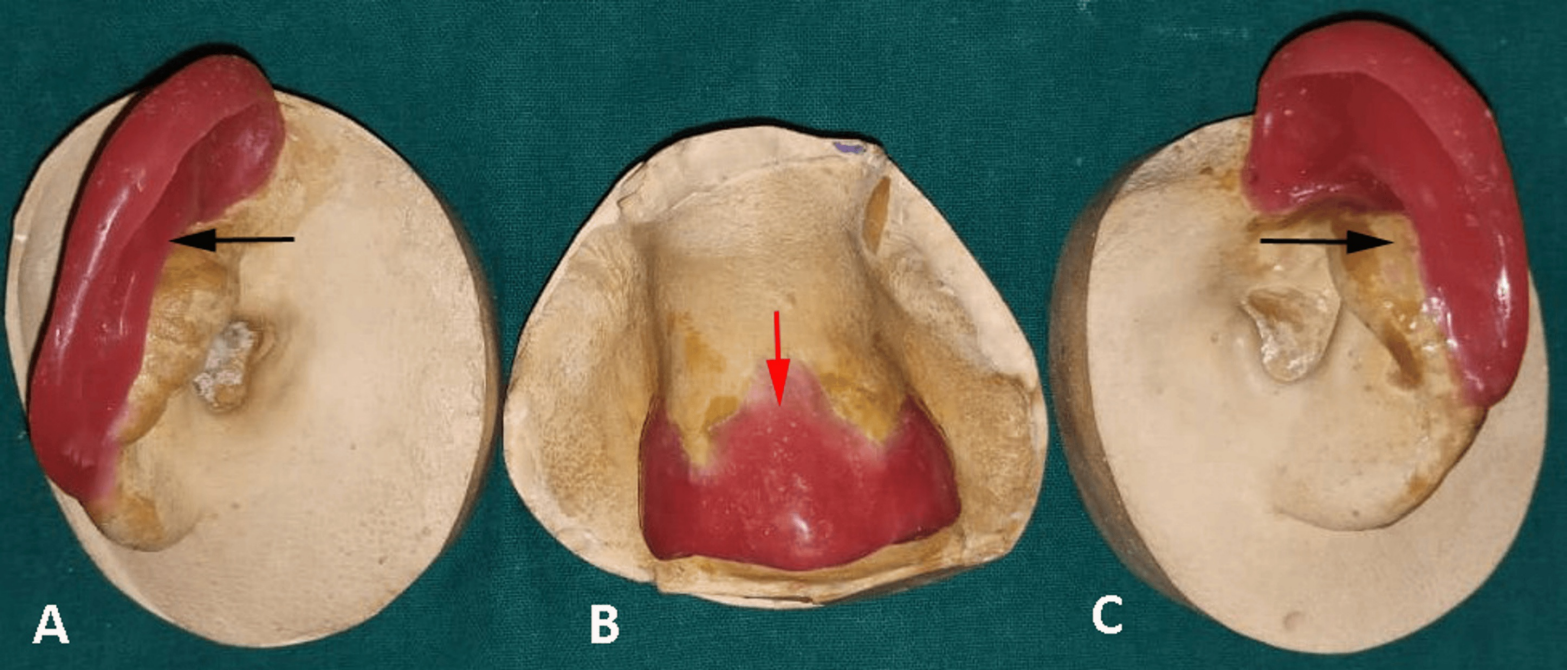
Wax pattern fabrication. (A) Right ear (black arrow). (B) Nose (red arrow). (C) Left ear (black arrow)

A donor ear of comparable size and contour was selected from the clinical literature as a sculptural reference. An impression of the donor auricle was obtained, and molten modeling wax (Hindustan Modeling Wax; Hindustan Dental Products, Hyderabad, India) was poured into the mold to fabricate a preliminary wax ear pattern (Figures 6A-6C). This pattern was subsequently adapted and individually sculpted on each auricular working cast to achieve bilateral symmetry and an appropriate anatomical orientation in harmony with the patient’s residual facial landmarks.

Nasal wax pattern fabrication

The nasal prosthesis wax pattern was developed through direct sculpting of the nasal defect working cast, with careful reference to facial proportion indices and available photographic documentation. The nasal tip projection, alar width, and dorsal height were iteratively refined to achieve natural harmony with the patient's facial features. A combined wax try-in of both auricular and nasal patterns was conducted on the patient to evaluate the bilateral fit, contour accuracy, and overall esthetic integration prior to mold fabrication.

Mold design and flasking procedures

For the auricular prosthesis, a three-piece mold design was employed to accommodate the complex anatomical undercuts inherent in the helical and antihelical structures of the external ear. This configuration enabled the accurate reproduction of all surface details while ensuring atraumatic retrieval of the cured silicone prosthesis without tearing or distortion. For the nasal prosthesis, a two-piece mold was utilized; during the initial flasking stage, putty impression material (Aquasil Putty; Dentsply Sirona) was carefully placed within the external nares to preserve the contour and patency of the nasal apertures, preventing occlusion of the nasal passages by the investing stone medium. Both wax patterns were invested in Type III gypsum prior to the flasking.

RTV silicone processing and intrinsic colorimetric matching

**Figure 7:**
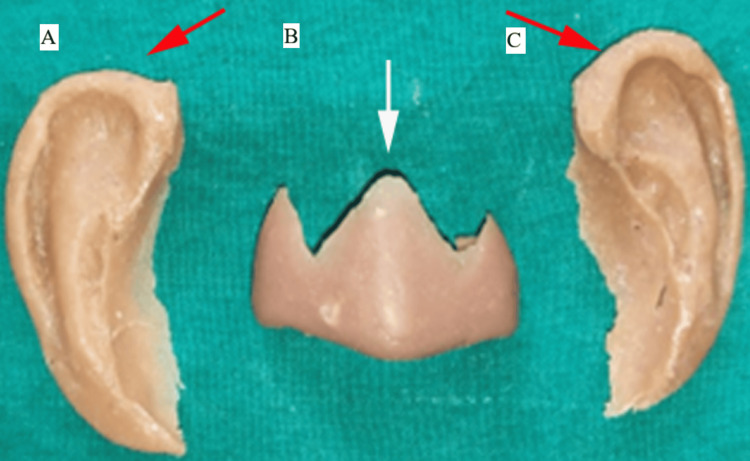
Silicon prosthesis of the (A) left and (C) right ears (red arrows), and (B) nose (white arrow)

Following complete dewaxing and mold preparation, the room-temperature vulcanizing (RTV) silicone elastomer was selected as the definitive material for both auricular and nasal prostheses because of its favorable biocompatibility, flexibility, hydrophobicity, and superior potential for intrinsic coloration. Color matching was achieved using an empirical pigment incorporation technique, wherein oxide-based silicone pigments were incrementally blended with the base silicone until a satisfactory match with the patient’s skin tone was obtained under standardized lighting conditions. Prior to packing, the mold surfaces were coated with soap solution as a separating medium. The intrinsically pigmented silicone was carefully packed into molds, and polymerization was allowed to occur at room temperature for 24 hours. Following complete curing, the prostheses were retrieved, excess flash was trimmed using fine scissors and a scalpel, and the surface characterization was refined as required (Figure 7).

Prosthesis retention and final delivery

**Figure 8:**
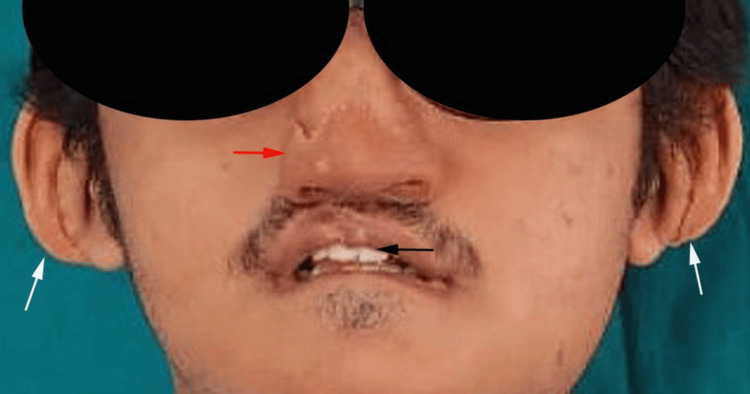
Postdelivery esthetics showing silicone ear prosthesis (white arrows), silicone nasal prosthesis (red arrow), and maxillary denture reducing maxillary hypoplasia (black arrow)

Bilateral auricular prostheses were designed for spectacle-frame retention, utilizing the patient's existing eyeglass frame as a noninvasive retention armature. This approach is particularly advantageous in adolescent patients given ongoing craniofacial growth and the impracticability of osseointegrated implants at this stage. The nasal prosthesis was retained using a medical-grade silicone adhesive applied to the peripheral tissue-contacting margins, with instructions for daily renewal. Complete dentures were inserted and evaluated for retention, stability, occlusal balance, and patient comfort, with minor adjustments completed chairside. Comprehensive oral and prosthetic hygiene instructions were provided to both the patient and his parent or guardian (Figure 8).

At the one-month follow-up evaluation, the patient demonstrated satisfactory adaptation to all prosthetic components. Intraoral denture retention and stability were assessed as clinically adequate, with isolated pressure points being identified and relieved. The patient reported subjective improvement in masticatory efficiency and speech intelligibility. Neither extraoral prosthesis exhibited visible surface degradation or colorimetric instability. At the three-, six-, and 12-month follow-up visits, the patient reported consistent use of the prosthesis and a notable improvement in social confidence, including the resumption of regular school attendance. The auricular prostheses retained satisfactory positional stability within the spectacle frames, and the nasal prosthesis adhesive interface remained intact with daily renewal, as instructed. No mucosal adverse reactions were identified at any of the tissue-contacting margins. The patient and guardian reported high satisfaction with the esthetic outcome and perceived it to be markedly superior to the pretreatment appearance. Clinically, the prostheses demonstrated satisfactory fit, retention, and integration with the surrounding facial tissues during follow-up examinations.

## Discussion

The present case demonstrates successful prosthodontic rehabilitation of a highly complex congenital craniofacial presentation characterized by the coexistence of complete edentulism, bilateral auricular agenesis, and nasal hypoplasia in an adolescent patient with CLP. Such a combination of intraoral and multiple extraoral defects in a single patient is exceedingly rare, and to the best of our knowledge, simultaneous rehabilitation using conventional complete dentures in conjunction with bilateral auricular and nasal prostheses has not been previously reported. This underscores the clinical significance and novelty of this case.

Prosthodontic rehabilitation in patients with CLP is inherently challenging due to maxillary hypoplasia, altered palatal morphology, scar tissue formation, and compromised denture-bearing areas, all of which adversely affect the retention, stability, and support of conventional prostheses [5]. In the present case, these anatomical limitations were compounded by complete edentulism at a young age. Despite these constraints, satisfactory functional and esthetic outcomes have been achieved through meticulous impression techniques, careful border molding, and appropriate occlusal rehabilitation, highlighting the continued relevance of conventional prosthodontic principles in complex clinical scenarios.

The decision to avoid implant-supported prostheses was based on multiple considerations. At 17 years of age, the patient was likely to have ongoing craniofacial growth, and implant placement at this stage may result in positional discrepancies over time owing to skeletal development [9]. Additionally, CLP-associated maxillary deficiencies and surgical scarring may limit the availability of adequate bone for implant placement and increase the surgical risk [10]. Financial constraints further influenced treatment planning, making conventional complete dentures pragmatic, reversible, and cost-effective. Previous reports have supported the use of implant-based rehabilitation in skeletally mature patients with CLP, suggesting that such modalities may be considered in the future once growth is complete [11].

Rehabilitation of extraoral defects using maxillofacial prostheses plays a crucial role in restoring facial form and improving psychosocial outcomes. In the present case, an RTV silicone elastomer was selected as the material of choice because of its favorable biomechanical properties, including flexibility, durability, biocompatibility, and ability to closely mimic skin texture and translucency [8]. Intrinsic coloration further enhanced the esthetic integration of prostheses with the surrounding tissues. These findings are consistent with the previous literature, emphasizing the superiority of silicone materials over acrylic alternatives for extraoral prosthetic applications.

Retention of the auricular prostheses was achieved using spectacle frames, which provided a noninvasive, cost-effective, and easily adjustable method. This approach is particularly advantageous in growing patients, where osseointegrated implants are contraindicated or deferred [6,12]. Similarly, the nasal prosthesis was retained using a medical-grade adhesive, ensuring adequate stability while maintaining patient comfort and ease of use. These retention strategies align with the established clinical protocols for managing congenital facial defects in resource-limited settings.

A notable technical aspect of this case was the use of a three-piece mold design for auricular prostheses, which facilitated accurate reproduction of complex anatomical undercuts and allowed atraumatic retrieval of the silicone prosthesis without distortion. Conventional two-piece molds may be inadequate for replicating intricate auricular structures, and the use of multipiece molds has been recommended in previous reports to enhance prosthetic accuracy [6,7]. The successful application of this technique in a bilateral setting contributes to its clinical relevance.

The outcomes observed in this case extended beyond functional rehabilitation. The patient reported marked improvements in masticatory efficiency, speech clarity, and social confidence following prosthetic intervention. These findings highlight the significant psychosocial benefits of comprehensive prosthodontic rehabilitation in patients with craniofacial anomalies, as emphasized in previous studies [3]. Restoration of facial appearance plays a pivotal role in reducing social stigma and improving quality of life in such individuals.

The constellation of bilateral CLP, bilateral auricular agenesis, nasal hypoplasia, and complete absence of permanent dentition in the present patient strongly suggests the possibility of an underlying craniofacial developmental syndrome. Differential diagnoses considered included Treacher Collins syndrome, Nager syndrome, and oculoauriculovertebral spectrum (Goldenhar syndrome), all of which may present with varying combinations of craniofacial asymmetry, auricular anomalies, mandibulofacial dysostosis, and clefting defects. However, the patient did not demonstrate several characteristic findings typically associated with these syndromes, such as limb anomalies in Nager syndrome [13], epibulbar dermoids or vertebral defects in Goldenhar syndrome [14], or the classic zygomatic and mandibular hypoplasia frequently observed in Treacher Collins syndrome [15]. Furthermore, the absence of comprehensive genetic evaluation, advanced imaging, and syndromic work-up limited the ability to establish a definitive diagnosis. The clinical presentation may, therefore, represent an atypical or overlapping craniofacial developmental spectrum disorder. Similar diagnostic challenges have been described in patients with complex craniofacial anomalies where phenotypic overlap and limited access to molecular testing preclude definitive syndromic classification.

Despite favorable outcomes, certain limitations must be acknowledged. Cone-beam computed tomography was not performed because of financial and resource limitations. This represents a major limitation of the present case, as the absence of radiographic evaluation precluded comprehensive assessment for unerupted or impacted permanent teeth, underlying alveolar pathology, and alveolar bone quality relevant to prosthetic rehabilitation. Comprehensive radiographic assessment should be considered essential in similar edentulous adolescent patients whenever feasible. In addition, audiological evaluation and assessment for bone-anchored hearing rehabilitation (BAHA) were not conducted despite the high likelihood of conductive hearing loss associated with bilateral auricular agenesis. Consequently, the patient’s hearing status and candidacy for hearing rehabilitation could not be determined. Future multidisciplinary follow-up should include comprehensive audiological assessment and BAHA evaluation, with consideration of the potential interaction between future hearing devices and the spectacle-frame retention system used for the auricular prostheses. Furthermore, although the constellation of craniofacial anomalies raised suspicion of an underlying syndromic condition, genetic consultation and chromosomal or gene panel testing were not performed because of financial and resource limitations. Future multidisciplinary management with longer follow-up, comprehensive audiological and genetic evaluation, and incorporation of digital technologies may further improve diagnostic accuracy and treatment outcomes. The reported improvements in masticatory efficiency and speech intelligibility were based on subjective patient and guardian feedback obtained during follow-up visits and routine clinical interaction, including improved ability to chew solid food, clearer conversational speech, and increased comfort during social communication. No validated objective assessment scales or standardized speech evaluation tools were used.

From a clinical perspective, this case reinforces that, even in the absence of advanced resources, a well-planned, multidisciplinary approach utilizing conventional prosthodontic techniques can achieve satisfactory functional and esthetic rehabilitation in patients with complex congenital craniofacial defects. These techniques are reproducible, cost-effective, and adaptable to similar clinical scenarios, particularly in resource-constrained settings.

## Conclusions

This case report describes the prosthodontic rehabilitation of a 17-year-old patient with CLP, complete edentulism, bilateral auricular agenesis, and nasal hypoplasia using conventional complete dentures and custom silicone auricular and nasal prostheses. At short-term follow-up, clinically observable improvement in facial esthetics and patient-reported improvement in mastication, speech, and social confidence were noted. The case highlights the feasibility of using conventional, noninvasive, and cost-effective prosthodontic techniques for complex craniofacial rehabilitation in resource-limited settings. However, the absence of standardized outcome assessment tools, comprehensive radiographic and audiological evaluation, genetic work-up, and long-term follow-up limits the generalizability of the findings. Further studies incorporating objective functional measures and larger patient cohorts are warranted.
